# A retrospective cost-analysis of additional homeopathic treatment in Germany: Long-term economic outcomes

**DOI:** 10.1371/journal.pone.0182897

**Published:** 2017-09-15

**Authors:** Julia K. Ostermann, Claudia M. Witt, Thomas Reinhold

**Affiliations:** 1 Institute for Social Medicine, Epidemiology and Health Economics, Charité - University Medical Center, Berlin, Germany; 2 University of Maryland School of Medicine, Center for Integrative Medicine, Baltimore, Maryland, United States of America; Johns Hopkins University Bloomberg School of Public Health, UNITED STATES

## Abstract

**Objectives:**

This study aimed to provide a long-term cost comparison of patients using additional homeopathic treatment (homeopathy group) with patients using usual care (control group) over an observation period of 33 months.

**Methods:**

Health claims data from a large statutory health insurance company were analysed from both the societal perspective (primary outcome) and from the statutory health insurance perspective (secondary outcome). To compare costs between patient groups, homeopathy and control patients were matched in a 1:1 ratio using propensity scores. Predictor variables for the propensity scores included health care costs and both medical and demographic variables. Health care costs were analysed using an analysis of covariance, adjusted for baseline costs, between groups both across diagnoses and for specific diagnoses over a period of 33 months. Specific diagnoses included depression, migraine, allergic rhinitis, asthma, atopic dermatitis, and headache.

**Results:**

Data from 21,939 patients in the homeopathy group (67.4% females) and 21,861 patients in the control group (67.2% females) were analysed. Health care costs over the 33 months were 12,414 EUR [95% CI 12,022–12,805] in the homeopathy group and 10,428 EUR [95% CI 10,036–10,820] in the control group (p<0.0001). The largest cost differences were attributed to productivity losses (homeopathy: EUR 6,289 [6,118–6,460]; control: EUR 5,498 [5,326–5,670], p<0.0001) and outpatient costs (homeopathy: EUR 1,794 [1,770–1,818]; control: EUR 1,438 [1,414–1,462], p<0.0001). Although the costs of the two groups converged over time, cost differences remained over the full 33 months. For all diagnoses, homeopathy patients generated higher costs than control patients.

**Conclusion:**

The analysis showed that even when following-up over 33 months, there were still cost differences between groups, with higher costs in the homeopathy group.

## Introduction

To date, the majority of German statutory health insurance companies reimburse homeopathy within the context of integrated care contracts [1]. Homeopathy consists of an extensive consultation and the prescription of homeopathic medicines. These homeopathic medicines are often diluted up to a concentration to which no molecule of the original substance can be found [2] and there is no valid scientific explanation for the mechanism of action of these diluted homeopathic medicines [2,3]. Because of this, critical voices are rising arguing that spending money on homeopathic treatments is a waste of money in times of limited resources for health care [4,5]. Furthermore, they see the reimbursement of homeopathic treatments statutory health insurance companies as an indirect legitimation of a non-scientific concept [5,6]. Yet, supporters of homeopathic treatment argue that individually prescribed homeopathic medicines are superior to placebo and quote respective meta-analyses [7], while critics quote those meta-analyses that come to the opposite conclusions [8].

Others explain the benefits by the fact that a homeopathic treatment consists of both the prescription of homeopathic medicines and a time-extensive consultation which allows to care more intensively for patients’ needs [9,10]. That homeopathic physicians take more time to treat their patients might at least partly explain the huge interest of patients in homeopathy and their demand for homeopathic treatments [11,12].

The integrated care contract ‘homeopathy’, this analysis is based on, is optional for both patients and physicians. Patients can subscribe to the integrated care contract at any time. Patients in the integrated care contract ‘homeopathy’ can receive homeopathic treatment in addition to usual care. Under the integrated care contract, the cost of the homeopathic treatment is covered by the patient’s insurance company; the patient receives the homeopathic treatment for free and the physician receives additional reimbursement. The evidence from economic evaluations of homeopathy is inconclusive: whereas some studies have shown that it offers cost-saving potential [13–15], other evaluations have shown it to be either more expensive [16–18] or to have similar costs [19] as usual care. A recent review by Viksveen concluded that the methodologies used in current studies are often weak and that it is therefore impossible to derive unequivocal conclusions from cost evaluations of homeopathy [20]. Our previous analyses of the integrated care contract ‘homeopathy’ showed that the cumulative costs of patients using homeopathic treatment in addition to usual care over an 18-month period were higher than the costs of patients using only usual care. However, the costs incurred by the two groups seemed to converge towards the end of the observation period [21] This finding emphasized the need for further analyses based on a longer observation period.

Therefore, the aim of the current study was to compare the health care costs of patients using homeopathic treatment in addition to usual care with the costs of patients using only usual care over a period of 33 months.

## Methods

### Study design and participants

We included male and female insureds with no age constraints. Patients were labelled as homeopathy patients if they subscribed to the integrated care contract homeopathy in 2011, regardless of whether they used homeopathy during the study. Subscription to the integrated care contract implied that the homeopathy patients had visited a physician in the first three months of the observational period. To level out a possible discrepancy in health care resource consumption between homeopathy patients and controls, control patients had to have visited a physician during the first three months of the study period. Moreover, control patients had to meet the following additional inclusion criteria: they did not subscribe to the integrated care contract homeopathy during the study, they were continuously insured by the health insurance company, and they were successfully matched with a homeopathy patient using propensity scores. For more details on the selection of patients, see Ostermann et al. [21]. In the long-term analysis discussed here, we followed up with patients from the previous analysis. However, patients who left the statutory health insurance company prior to 18 months (i.e., before the end of the previous study) were excluded from the analyses. In addition to analysing the health care costs of all patients, independently from a specific diagnosis, we also subdivided patients into the following physician-confirmed diagnosis-groups: depression (ICD-10 F32), migraine (G43), allergic rhinitis (J30), allergic asthma (J45), atopic dermatitis (L20), and tension headache (R51). Cost analysis of these subgroups was not limited to the specific costs associated with the disease.

We followed the guidelines for secondary data analyses. De-identified health claims data were provided by the statutory health insurance company. We had no key for de-identifying the data. This study was approved by the Ethics Committee of the Charité - Universitätsmedizin Berlin (EA2/121/12).

### Propensity score matching

Because subscription to the integrated care contract homeopathy was open to all insureds of the statutory health insurance company, we conducted an observational analysis. To balance baseline characteristics and minimize selection bias of patients between groups, homeopathy and control patients were matched 1:1 using propensity scores [22]. For patients in both groups, propensity scores for the outcome ‘user of the integrated care contract’ (yes/no) were calculated. Propensity scores were computed using the following covariates: sex (male/female), age (continuous), comorbidities (disease present, yes/no), cumulative different unit costs one year prior to the study period (continuous), length of stay in a hospital (continuous), days of sick leave (continuous) and statutory sick pay costs (continuous), duration of outpatient rehabilitation (continuous), level of care intensity (‘1’, ‘2’, ‘3’ or ‘3 plus’), disease-management-program participation (yes/no), usage of GP-centred care (yes/no) and population density (inhabitants per square kilometre, continuous). A caliper width of 0.25 of the standard deviation of the logit of the propensity score was applied to match control patients to homeopathy patients. As seasonal effects could bias patients’ characteristics and costs, the matching process was performed separately for each quarter of the year 2011. For homeopathy patients, the index date of the observational period was their subscription date to the integrated care contract. Cumulative costs one year prior to the study period were therefore calculated from the index date over a period of 12 months. For the control patients, the index date of the matched homeopathy patients, which was required for the calculation of the propensity score, was known only after the matching process was completed. To overcome this obstacle, the index date for the controls was specified as the middle of the quarter of the respective matching process. For each of the four matching groups, health claims data were analysed for a total of 45 months, including 12 months before and 33 months after the respective index date. Data were therefore analysed from January 2010 to September 2014.

### Economic analysis

Health care costs were analysed from the societal perspective across diagnoses (primary analysis) and between diagnoses (secondary analysis). Additionally, costs were analysed from the perspective of the statutory health insurance company. To factor in the productivity loss from the societal perspective, we adopted the human capital approach with a daily mean gross income of EUR 239.20 and a cut-off period of six weeks [23]. A cut-off period of six weeks was used because, in Germany, the employer continues to pay the employee’s salary in case of illness up to six weeks. Statutory sick pay from the statutory health insurance is paid only after six weeks. Costs were not discounted. Negative costs in the dataset that could have arisen due to accounting reasons from previous reimbursement periods were set at zero to avoid interpretation issues. To monitor the source of costs for outpatient care, outpatient costs were divided into costs generated by homeopathic physicians who participated in the integrated care contract homeopathy and other physicians. Controls could consult homeopathic physicians; however, they could only do so outside the framework of the integrated care contract.

### Statistical analysis

For the primary endpoint, cumulative health care costs were analysed from a societal perspective across diagnoses and between groups after 33 months. We performed an analysis of covariance (ANCOVA), controlling for each group’s respective baseline costs (cumulative costs from month -12 until month 0). Total costs consisted of outpatient care costs (generated by homeopathic physicians and by other physicians), medication costs, productivity losses, costs of the integrated care contract, inpatient costs and other costs. Total costs and single cost types were added for the period of 12 months before the start of the observational period (month -12 to month 0) and for the subsequent 33 months. Costs incurred after the start of the observational period were divided into three-month intervals (months 1–3, 4–6, 7–9, 10–12, 13–15, 16–18, 19–21, 22–24, 25–27, 28–30, 31–33). Costs were compared between groups and between diagnoses using ANCOVAs, with the respective baseline cost values as the covariates. To assess how the cumulative total costs developed over time, cost progression over the observation period was analysed between groups and across diagnoses, from both the societal and statutory health insurance perspectives. The test for the primary end point was two-sided with a significance level of 0.05. All other tests were exploratory and were two-sided with a significance level of 0.05. SAS software version 9.3 (SAS Institute Inc., Cary, U.S.) was used for participant matching. The analyses were computed following a pre-specified statistical analysis plan and using R version 3.1.0 [24].

## Results

Out of all 22,275 patients per group from the previous study, 336 patients (1.5%) from the homeopathy group and 414 patients (1.9%) from the control group left the statutory health insurance company. Therefore, 21,939 patients (67.4% female) in the homeopathy group and 21,861 patients (67.2% female) in the control group could be analysed. The sample was quite comparable in terms of baseline characteristics (Table 1).

**Table 1:** Baseline characteristics of all patients. Data include the mean (SD) and the number of persons (%).

	Homeopathy (n = 21939)	Control (n = 21861)
Women (n)	14779 (67.4)	14969 (67.2)
Age (years)	33.86 (20.0)	34.2 (20.1)
Sick leave days previous 12 months	9.61 (35.38)	10.19 (36.45)
Hospital cases previous 12 months	0.22 (0.69)	0.21 (0.64)
Cumulated costs previous 12 months		
Total	1841 (5355)	1846 (5140)
Medication	300 (2882)	314 (2444)
Inpatient	552 (3059)	552 (3147)
Diagnosis (n)		
Other	13261 (60.4)	13228 (60.5)
Depressive disorder (F33)	3033 (13.8)	3018 (13.8)
Migraine (G43)	880 (4.0)	872 (4.0)
Allergic rhinitis (J30)	1128 (5.1)	1119 (5.1)
Asthma (J45)	1229 (5.6)	1222 (5.6)
Atopic dermatitis (L20)	1456 (6.6)	1460 (6.7)
Headache (R51)	952 (4.3)	942 (4.3)
State of residence (n)		
Abroad	35 (0.2)	24 (0.1)
Baden-Wuerttemberg	3203 (14.6)	2234 (10.2)
Bavaria	2881 (13.1)	2388 (10.9)
Berlin	2222 (10.1)	1934 (8.9)
Brandenburg	427 (2.0)	534 (2.4)
Bremen	275 (1.3)	171 (0.8)
Hamburg	1253 (5.7)	1014 (4.6)
Hesse	1844 (8.4)	1995 (9.1)
Mecklenburg-Western Pomerania	232 (1.1)	380 (1.7)
Lower Saxony	2094 (9.5)	2281 (10.4)
North-Rhine Westphalia	4097 (18.7)	5719 (26.2)
Rhineland-Palatinate	870 (4.0)	954 (4.4)
Saarland	196 (0.9)	203 (0.9)
Saxony	406 (1.9)	432 (2.0)
Saxony-Anhalt	131 (0.6)	299 (1.4)
Schleswig-Holstein	1486 (6.8)	1021 (4.7)
Thuringia	287 (1.3)	278 (1.3)

Costs from month -12 to month 0 were comparable between homeopathy patients and controls (societal perspective: homeopathy EUR 3666 [3549–3783]; controls EUR 3769 [3654–3884]; statutory health insurance perspective: homeopathy EUR 1841 [1770–1912]; controls EUR 1846 [1777–1914]).

The adjusted mean costs after 33 months were EUR 12,414 [95% CI 12,022–12,805] in the homeopathy group and EUR 10,429 [10,037–10,821] in the control group (mean difference EUR 1985 [1946–2024], p<0.0001). Productivity loss (homeopathy EUR 6,289 [6,118–6,460]; controls EUR 5,498 [5,326–5,670]; mean difference: EUR 791 [765–817], p<0.0001) and outpatient care (homeopathy EUR 1,794 [1,770–1,818]; controls EUR 1,438 [1,414–1,462]; mean difference: EUR 356 [346–366], p<0.0001) accounted for the majority of total costs (Table 2). Subtracting productivity loss from total costs revealed a cost difference of EUR 1,130 [803–1,457] (homeopathy EUR 6,093 [5,766–6,420]; controls EUR 4,963 [4,635–5,290]). In both groups, outpatient care costs were predominantly generated by other physicians (homeopathy EUR 1,531 [1,501–1,561]; controls EUR 1,355 [1,325–1,384], p<0.0001). Apart from treating homeopathy patients, physicians who participated in the integrated care contract (‘homeopathic physician’) could treat control patients outside the framework of the integrated care contract with either homeopathy or conventional medicine. Homeopathic physicians only generated approximately 13% of outpatient care costs in the homeopathic group (EUR 230 [227–233]) and 3% of outpatient care costs in the control group (EUR 39 [36–42], p<0.0001).

**Table 2:** Adjusted means for different cost types over 33 months after the start of the integrated care contract for all patients, societal perspective.

	Homeopathy (n = 21939)		Control (n = 21861)		
Type of cost	N Cost utilization	Adj. mean (EUR) (95% CI)	N Cost utilization	Adj. mean (EUR) (95% CI)	p-value
Integrated care contract	21,938	299 (297–302)	-	-	<0.0001
Outpatient	21,939	1,794 (1,770–1,818)	21,861	1,438 (1,414–1,462)	<0.0001
Homeopathic physician	21,386	230 (227–233)	948	39 (36–42)	<0.0001
Other physician	21,751	1,531 (1,501–1,561)	21,861	1,355 (1,325–1,384)	<0.0001
Medication	20,614	1,461 (1,209–1,713)	20,907	1,069 (816–1,321)	0.031
Productivity loss	9,450	6,289 (6,118–6,460)	9,528	5,498 (5,326–5,670)	<0.0001
Inpatient	6,831	1,674 (1,593–1,755)	6,527	1,485 (1,404–1,567)	0.001
Other	28,266	106 (103–109)	24,291	102 (99–105)	0.031
**Total**	**21,939**	**12,414 (12,022–12,805)**	**21,861**	**10,429 (10,037–10,821)**	**<0.0001**

Across all diagnoses, costs for homeopathy patients were higher than costs for control patients over the period of 33 months. The range of costs varied greatly across diagnoses, with the highest costs being generated by patients with depression (homeopathy (n = 3,033), EUR 25,107 [95% CI 24,131–26,083]; controls (n = 3,018), EUR 21,892 [20,913–22,870], p<0.0001), and the lowest costs generated by patients with atopic dermatitis (homeopathy (n = 1,456) EUR 7,425 [6,894–7,955]; controls (n = 1,460), EUR 6,123 [5,593–6,653], p = 0.001). However, this difference among groups was not always statistically significant (Table 3).

**Table 3:** Adjusted means for different cost types and diagnoses over 33 months after the beginning of the integrated care contract, societal perspective.

			Homeopathy		Control		
	Type of cost		N Cost utilization	Adj. mean (EUR) (95% CI)	N Cost utilization	Adj. mean (EUR) (95% CI)	p-value
**Depression** **n = 6051**	Integrated care contract		3,033	364 (357–371)	-	-	<0.0001
	Outpatient		3,033	3,228 (3,150–3,306)	3,018	2,607 (2,529–2,686)	<0.0001
		Homeopathic physician	2,944	297 (286–308)	153	63 (51–74)	<0.0001
		Other physicians	3,026	2,780 (2,706–2,854)	3,018	2,455 (2,380–2,529)	<0.0001
	Medication		2,937	2,099 (1,846–2,353)	2,970	1,965 (1,711–2,219)	0.464
	Productivity loss		1,891	14,741 (13,994–15,489)	1,786	12,808 (12,058–13,557)	<0.0001
	Inpatient		1,387	3,070 (2,822–3,317)	1,262	2,738 (2,489–2,986)	0.064
	Other		5,392	195 (182–207)	4,686	191 (178–203)	0.635
	**Total**		**3,033**	**25,107 (24,131–26,083)**	**3,018**	**21,892 (20,913–22,870)**	**<0.0001**
**Migraine** **n = 1752**	Integrated care contract		880	329 (318–341)	-	-	<0.0001
	Outpatient		880	1,981 (1,881–2,081)	872	1,653 (1,553–1,753)	<0.0001
		Homeopathic physician	849	239 (228–251)	33	38 (26–49)	<0.0001
		Other physicians	878	1,652 (1,557–1,747)	872	1,549 (1,453–1,644)	0.132
	Medication		842	944 (772–1,116)	851	992 (820–1,165)	0.695
	Productivity loss		549	8,337 (7,401–9,273)	546	7,560 (6,619–8,500)	0.251
	Inpatient		312	2,321 (1,616–3,026)	294	1,803 (1,094–2,511)	0.310
	Other		1,315	95 (85–106)	1,125	85 (75–96)	0.190
	**Total**		**880**	**14,721 (13,316–16,126)**	**872**	**12,797 (11,385–14,208)**	**0.058**
**All. Rhinitis** **n = 2247**	Integrated care contract		1,128	325 (315–335)	-	-	<0.0001
	Outpatient		1,128	1,646 (1,570–1,722)	1,119	1,307 (1,231–1,383)	<0.0001
		Homeopathic physician	1,096	234 (223–244)	50	29 (19–39)	<0.0001
		Other physicians	1,122	1,349 (1,277–1,422)	1,119	1,222 (1,150–1,295)	0.015
	Medication		1,069	1,066 (829–1,303)	1,090	786 (548–1,024)	0.103
	Productivity loss		537	5,427 (4,796–6,058)	539	4,652 (4019–5,286)	0.090
	Inpatient		302	1,097 (929–1,264)	320	934 (766–1,102)	0.177
	Other		1,441	78 (68–88)	1,233	73 (63–82)	0.475
	**Total**		**1,128**	**10,222 (9,390–11,055)**	**1,119**	**8,352 (7,516–9,188)**	**0.002**
**Asthma** **n = 2451**	Integrated care contract		1,229	319 (308–329)	-	-	<0.0001
	Outpatient		1,229	1,920 (1,789–2,051)	1,222	1,579 (1,448–1,711)	<0.0001
		Homeopathic physician	1,195	241 (230–253)	46	51 (40–62)	<0.0001
		Other physicians	1,224	1,635 (1,427–1,843)	1,222	1,528 (1,319–1,736)	0.476
	Medication		1,206	1,488 (1,260–1,716)	1,209	1,271 (1,043–1,500)	0.187
	Productivity loss		526	5,808 (5,190–6,426)	515	4,605 (3,986–5,225)	0.007
	Inpatient		421	1,719 (1,402–2,036)	381	1,696 (1,378–2,014)	0.920
	Other		1,692	107 (94–121)	1,432	110 (96–123)	0.807
	**Total**		**1,229**	**12,178 (11,238–13,118)**	**1,222**	**10,145 (9,202–11,088)**	**0.003**
**Atopic dermatitis** **n = 2916**	Integrated care contract		1,456	282 (274–290)	-	-	<0.0001
	Outpatient		1,456	1,533 (1,439–1,626)	1,460	1,186 (1,092–1,279)	<0.0001
		Homeopathic physician	1,429	206 (199–213)	68	38 (31–45)	<0.0001
		Other physicians	1,452	1,312 (1,165–1,459)	1,460	1,095 (949–1,242)	0.041
	Medication		1,416	806 (684–929)	1,436	734 (612–856)	0.411
	Productivity loss		455	3,201 (2,830–3,572)	423	2,555 (2,184–2,926)	0.016
	Inpatient		398	897 (752–1,042)	383	868 (723–1,013)	0.784
	Other		1,662	87 (79–94)	1,451	84 (76–91)	0.579
	**Total**		**1,456**	**7,425 (6,894–7,955)**	**1,460**	**6,123 (5,593–6,653)**	**0.001**
**Headache** **n = 1894**	Integrated care contract		952	289 (279–300)	-	-	<0.0001
	Outpatient		952	1,964 (1,857–2,070)	942	1,484 (1,377–1,591)	<0.0001
		Homeopathic physician	926	207 (197–217)	39	41 (31–51)	<0.0001
		Other physicians	950	1,669 (1,567–1,770)	942	1,374 (1,272–1,475)	<0.0001
	Medication		911	952 (736–1,168)	922	976 (759–1,192)	0.879
	Productivity loss		444	7,219 (6,336–8,102)	440	6,183 (5,295–7,071)	0.105
	Inpatient		332	1,604 (1,225–1,984)	328	1,734 (1,353–2,115)	0.637
	Other		1,388	95 (86–104)	1172	92 (83–101)	0.668
	**Total**		**952**	**12,733 (11,528–13,937)**	**942**	**11,328 (10,118–12,539)**	**0.107**

The number of homeopathy patients who generated costs under the integrated care contract during months 1–3 was 100% (n = 21,928). During months 4–6 the number of patients generating costs dropped to 56% (n = 12,331). At the end of our previous study, in months 16–18, only 26% (n = 5,782) of the homeopathy patients still generated costs under the integrated care contract. Five months later, during months 31–33 (the end of our current study), this number continued to drop to 20% (n = 4,326).

**Fig 1:**
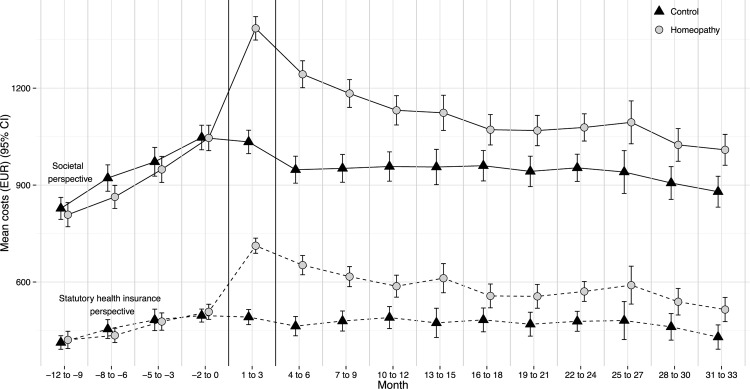
Mean overall cost (EUR) progression by group from the societal and statutory health insurance perspectives from month -12 until month 33. Error bars denote 95% CIs. Months 1 to 3 indicate the start of the integrated care model. Costs from month 1 onward are adjusted to baseline costs (month -12 to month 0).

**Fig 2:**
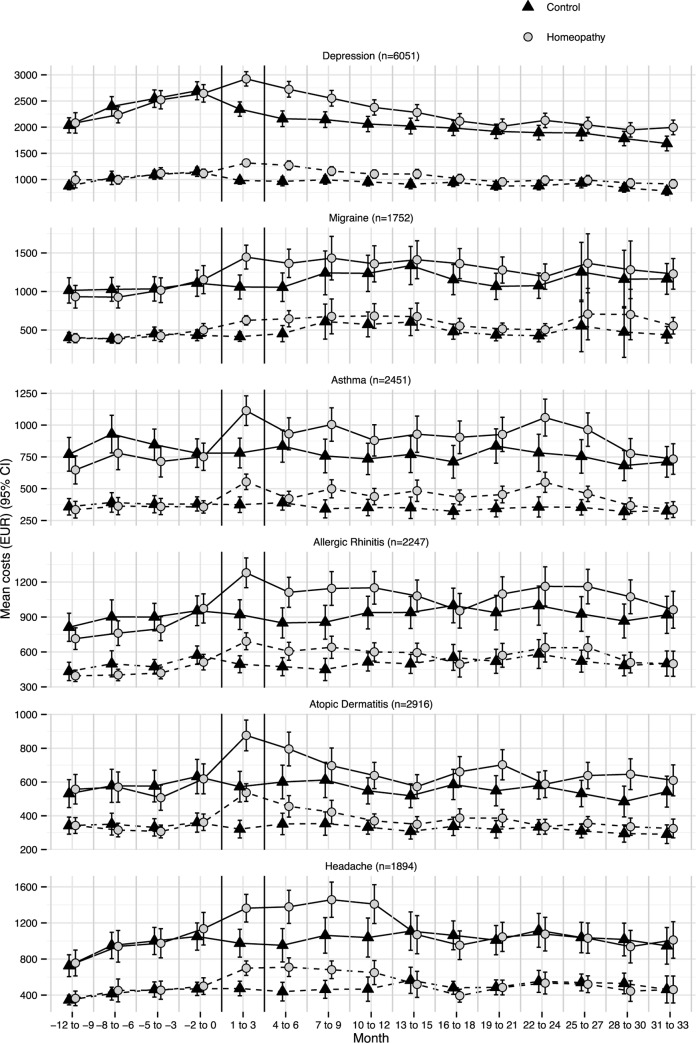
Mean overall cost (EUR) progression by group and diagnosis from the societal (solid line) and statutory health insurance (dashed line) perspectives from month -12 until month 33. Error bars denote 95% CIs. Months 1 to 3 indicate the start of the integrated care model. Costs from month 1 onward are adjusted to baseline costs (month -12 to month 0).

Total costs from the societal perspective were two times higher than total costs from the statutory health insurance perspective (homeopathy EUR 6,507 [6,173–6,841]; controls EUR 5,199 [4,865–5,534], p<0.0001), with similar cost progression in both groups. Higher costs from the societal perspective were mainly driven by indirect costs, i.e., productivity loss. The costs from the statutory health insurance perspective, due to patients’ inability to work and statutory sick pay, accounted for approximately 10% of productivity loss. The greatest cost difference was observed in months 1 to 3, which coincides with the beginning of the integrated care contract and with adjusted mean costs of EUR 115 [115–115] (from both perspectives) in the homeopathic group. After month three the cost difference between the groups decreased. However, a cost difference between the groups persisted beyond month 18 –the end of the previous study–and continued until the end of this study’s observation time, i.e., month 33 (Fig 1). The cost difference between the groups from month 18 until month 33 remained relatively steady. The cost progressions within the specific diagnoses were generally similar to the cost progressions across diagnoses. Greater variations in the data were due to smaller numbers of subjects. The cost progression of patients with headache (n = 1894) is an exception. A deeper analysis of headache patients revealed that a cost difference persisted only from months 1 to 12. After month twelve, no difference in costs between the groups could be observed (Fig 2).

## Discussion

### Key results

A comparison of adjusted health care costs showed that the total costs and unit costs of patients using homeopathy after 33 months of observation under the integrated care contract were higher than the costs for patients not using homeopathy. Cost differences between these two groups persisted throughout the observation period, from month 0 until month 33. Cost differences between groups decreased from month 0 until month 18. From month 18 until month 33, the cost progression of both groups ran almost parallel, although at different levels.

### Strengths and limitations

A strength of the current analysis is the large sample size. Using health claims data from a large statutory health insurance company allowed us to analyse 43,800 insureds over a period of nearly four years. Additionally, our main result, that the costs of homeopathy patients were higher than the costs of controls, remains constant across all sub-diagnoses and between the statutory health insurance and societal perspectives. This strengthens our confidence in our results. Moreover, the data were not restricted to a specific geographic area because the statutory health insurance company has clients throughout Germany. However, using data from only one statutory health insurance company limits the generalizability of the results. Clients of this statutory health insurance tend to be higher earners and better educated than clients using other German statutory health insurance companies. Using the propensity-score matching approach, we were able to generate good comparability between the groups but only in terms of the specific variables that influenced the propensity score. A limitation of this study is that it only analysed costs. Therefore, it is not possible to make conclusions about the outcomes of the integrated care contract. As every client of the statutory health insurance could potentially participate in the integrated care contract, a prospective randomized controlled study design was not possible. Therefore, an observational study design was used instead, which could have resulted in selection bias regarding the patients. Using the propensity score approach, we could only control for variables that were available in the health claims data. As we did not have information about such variables as social status or health consciousness, we cannot rule out the possibility that the groups were dissimilar in these characteristics. The homeopathic physicians who took part in the integrated care contract were both conventionally and homoeopathically trained and could provide both types of treatment within the health insurance system. The health claims data did not include any information about the type of treatment that generated common outpatient costs. Therefore, we were unaware which type of treatment, homeopathy or conventional medicine, the homeopathic physician offered to homeopathy patients or controls, apart from the specifications of the integrated care contract.

### Interpretation

Long-term analyses of studies are important because many studies have only a short-term observational period despite the fact that some consequences of health interventions might only be detectable long-term. In an observational study examining the effects and costs of homeopathic treatment in children with atopic eczema, homeopathic treatment was not superior to conventional treatment, but it was associated with higher costs after 12 months [17]. A follow-up analysis of the patients over 36 months showed that outcomes for homeopathy patients were not superior to outcomes for conventionally treated patients and that costs for homeopathy patients were still twice as high as costs for conventionally treated patients [18]. Our previously published short-term economic evaluation of the homeopathy integrated care contract showed that adjusted total costs were higher in the homeopathy group compared to the usual care group [21]. However, this cost difference seemed to decrease by month 18, the end of the observational study period [21]. To better understand these developments over time, we decided to lengthen the observation period. This longer study showed that even after 33 months a relevant cost difference persisted.

Health claims data make it possible to perform long-term analyses with relative ease, as the data are already collected for administrative purposes. Compared to collecting and analysing primary data, re-analysing secondary data is economical in terms of both time and money. We do not have data on the number of patients in the homeopathy group who remained subscribed to the integrated care contract for 33 months. However, the data do reveal the number of patients who generated costs during each three-month interval under the integrated care contract. The number of patients in the homeopathy group who generated costs under the integrated care contract decreased rapidly from the start of the observation period until months 4–6 and continued to drop until the end of the observation period, at which point only a fifth of the homeopathy patients were still generating costs under the integrated care contract. The total costs generated at the end of our previous study might therefore not be attributable entirely to the integrated care contract. The long-term cost difference might be attributable to the higher health consciousness of patients who voluntarily subscribe to an integrated care contract compared to control patients. As discussed previously, a visit to a homeopathic physician, especially the initial contact between physician and patient, could generate an extensive period of physician-patient communication. This might initiate further treatments and consultations with additional physicians [21]. In our short-term observation period, a comparable number of patients in each group had a mental diagnosis at baseline. However, at month 1–3, 8,660 (38.9%) more mental illnesses had been diagnosed in the homeopathy group compared to the control group. As mental illnesses are chronic conditions, treatment should continue over a long period of time. Therefore, contact with health services–and associated costs–could increase among these patients [25–27]. This might partially explain the cost difference between the groups over 33 months.

### Conclusion

These long-term cost analyses have produced similar results to previous short-term analyses. After nearly three years, the costs of patients who received homeopathy in addition to usual care were still higher than the costs of patients receiving only standard care.
